# Supplementary material for: Combined effects of age and BMI are related to altered cortical thickness in adolescence and adulthood
Source: Dev Cogn Neurosci. 2019 Nov 5;40:100728. doi: 10.1016/j.dcn.2019.100728 (PMC6913515; doi:10.1016/j.dcn.2019.100728)
Supplement: Supplementary file 1 [file mmc1.docx]

**Supplementary material: Combined effects of age and BMI are related to altered cortical thickness in adolescence and adulthood**

Margaret L. Westwater, Raquel Vilar-López, Hisham Ziauddeen, Antonio Verdejo-García, & Paul C. Fletcher

**Methods**

*Participants.* Lean adolescents were classified as those with 5^th^ ≤ age-adjusted BMI < 85^th^ percentile; overweight adolescents were those with 85^th^ ≤ age-adjusted BMI < 95^th^ percentile; and obese adolescents had adjusted BMI scores ≥ 95^th^ percentile. In the adult group, lean individuals were identified by 18.5 ≤ BMI ≤ 24.9 kg/m^2^; overweight adults 25.0 ≤ BMI ≤ 29.9 kg/m^2^; and obese adults ≤ 30.0 BMI < 40.0 kg/m^2^, the threshold for morbid obesity. To confirm the absence of metabolic comorbidities, blood samples were acquired from a subset (n = 43) of adolescents and assayed for insulin and total cholesterol levels. Samples were collected between 8.00 and 10.00 following an overnight fast.

*Structural MRI Analyses.* Structural MRI data were analysed using FreeSurfer (v 6.0.0, <https://surfer.nmr.mgh.harvard.edu/>) software. Briefly, the FreeSurfer pipeline first corrects MR images for magnetic field inhomogeneities. Normalized intensity images are created, affine-registered to the Talairach atlas (Talairach & Tournoux, 1988) and skull-stripped to remove non-brain tissue. In the preliminary segmentation, WM voxels are identified by their location, their intensity and the intensity of their adjacent voxels. The two hemispheres are separated, and WM voxels are grouped using a six-neighbours connected components algorithm, thus creating a single-filled volume for each hemisphere. A mesh of triangular faces is fitted to the resulting surface, with two triangles for each exposed voxel face, and it is then smoothed to reduce distortions. The initial smoothing procedure employs an algorithm that accounts for the local intensity of the original images (Dale & Sereno, 1993) at a higher spatial resolution, using trilinear interpolation. Correction for topological defects described by Fischl et al. (2001) ensures that the surface maintains the same topological properties as a sphere.

Following this initial reconstruction of the surface, a second iteration of smoothing is applied, which results in a realistic representation of the GM/WM boundary. This surface, called the white surface, is then deformed outwards toward the point where tissue contrast is maximal, maintaining the original constraints on smoothness and possibility of self-intersection (Fischl & Dale, 2000). This surface is referred to as the pial surface, as it represents the grey matter boundary that sits below the pia mater. Complete cortical surface reconstructions were visually inspected for quality control purposes and discarded when necessary.

Surfaces were then parcellated into distinct cortical regions using an automated procedure (Fischl et al., 2004). Within each subject, the cortical surface was transformed to a sphere in order to establish a coordinate system on the ‘unfolded’ surface (Fischl, Sereno, & Dale, 1999). Each vertex was assigned an anatomical label based on: 1) the probability that a given vertex belongs to a certain label, using a Bayesian approach; 2) mean curvature information of the region; and 3) contextual information, which, for each vertex, considers the labels that were assigned to neighbouring vertices. This labelling process iterates until the label assignment of each vertex no longer changes. We used the parcellation scheme developed by Desikan et al. (2006), which includes labels for gyri, sulci and 66 regions of interest. Following this, cortical thickness values for each ROI were calculated as the average distance between the white and pial surface within the region. Calculation of surface area was completed in the subjects’ native space, and values were defined as the sum of the areas of each triangular face located within the ROI. Global CT and SA values in each hemisphere were calculated as averages and summed totals of the ROIs, respectively.

*Quality Assurance.* Surface reconstructions were visually assessed by a blind rater (MLW) and classified according to a 5-point Likert scale (1 = poor, 2 = adequate poor, 3 = adequate, 4 = adequate good, 5 = good), where ‘poor’ or ‘adequate poor’ reconstructions were excluded from further analysis. As previous research suggests manual editing to be an appropriate quality assurance step for reconstructions rated ‘adequate’ or better (Backhausen et al., 2016), which does not significantly bias cortical thickness, surface area or white matter volume estimates (Ronan et al., 2016), MLW performed minimal, manual editing of the remaining surface reconstructions.


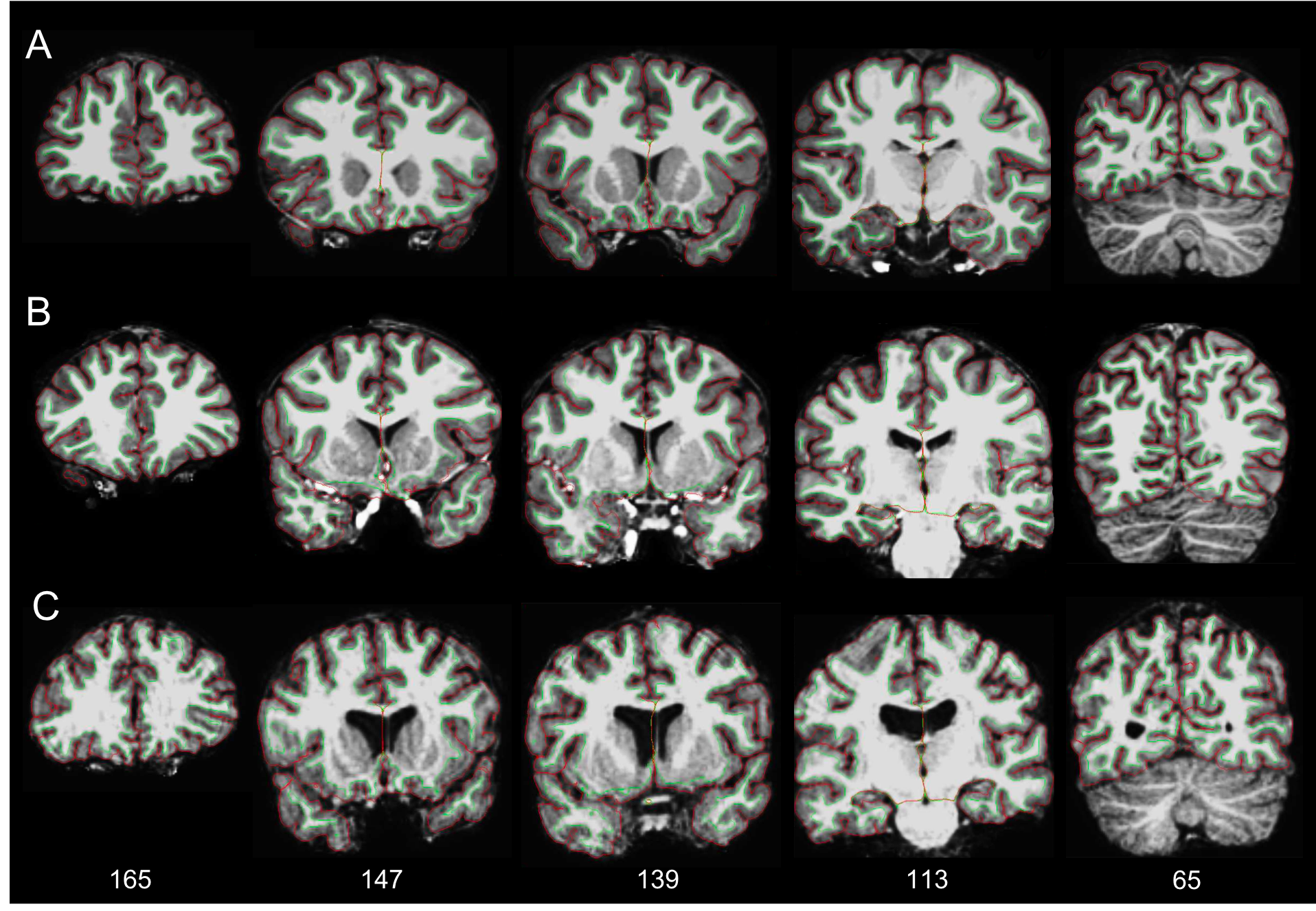
The following features were assessed when rating each reconstruction: overall resolution of the image, inclusion of all lobes, grey and white matter tissue contrast, artefacts (e.g., ghosting, ringing or Gibbs phenomenon, susceptibility), presence of non-brain tissue (e.g., inclusion of dura mater within the grey matter boundary) and the accuracy of grey and white matter boundaries. An example of ‘good’, ‘adequate’ and ‘poor’ surface reconstructions is shown in Supplementary Figure 1. In brief, ‘good’ and ‘adequate good’ reconstructions had high spatial resolution, with crisp boundaries of sub-cortical regions; a high contrast to noise ratio, enabling accurate segmentation of grey and white matter across the entire brain; no motion or ghosting artefacts and minimal susceptibility artefacts in known problem regions (e.g., medial temporal lobes); little to no non-brain tissue and few inaccuracies in the white matter boundary. The distinction between ‘good’ and ‘adequate good’ ratings reflected the degree of editing that would be required to achieve the ideal surface; ‘good’ reconstructions required little to no editing. ‘Adequate’ ratings were assigned to reconstructions, such as Supp. Figure 1B, that had adequate resolution and tissue contrast, minor susceptibility or motion artefacts, minimal inclusion of non-brain tissue and/or some inaccuracies in the white matter boundary that could be corrected via control points or manual addition/removal of white matter voxels. Finally, reconstructions with low resolution, significant ringing or ghosting artefacts, low tissue contrast that impeded accurate segmentation of grey and white matter and/or exclusion of portions of the brain were rated as either ‘poor’ or ‘adequate poor’ (see Supp. Figure 1C).

**Supplementary Figure 1.** Example FreeSurfer surface reconstructions with A) good, B) adequate and C) poor ratings. Coronal slices are shown in left = right orientation. Slice number is depicted below panel C.

Within the adolescent group, 2 (2.7%) reconstructions were of poor quality, 1 (1.4%) was adequate poor, 7 (9.6%) were adequate, 19 (26%) were adequate good and 44 (60.3%) were of good quality. Surface reconstructions within the adult group were rated: 2 (2.6%) poor, 1 (1.3%) adequate poor, 10 (12.8%) adequate, 35 (44.9%) adequate good and 30 (38.5%) good. We discarded three, poor-quality MRI scans from the adolescent group, and a fourth adolescent was excluded because of a corrupt file. Three adult structural scans were discarded for quality concerns. In total, 70 adolescents and 75 adults with high-quality structural scans were included in the CT analyses.

**
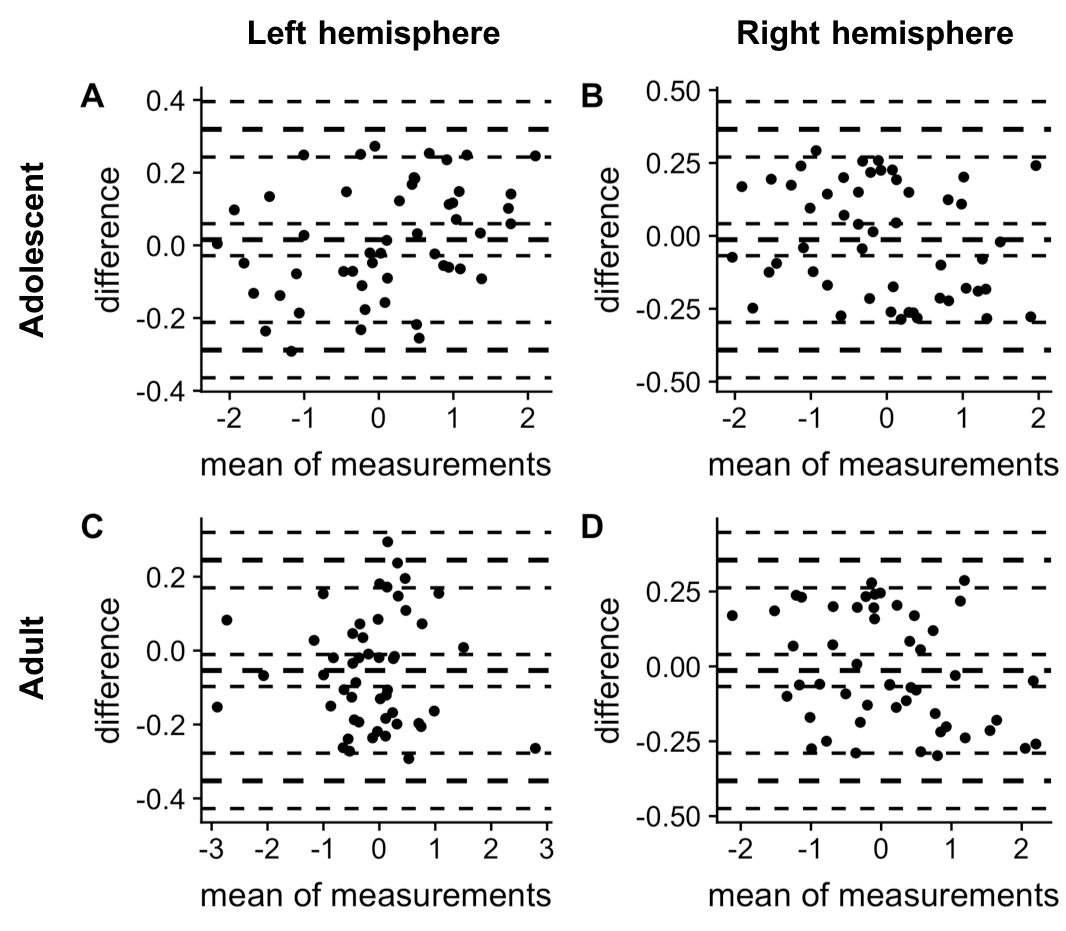
**Bland and Altmann plots (Supplementary Figure 2) were used to assess potential measurement bias from quality assurance procedures. The mean difference in cortical thickness approximated zero for adolescent left (M, SD = -0.01, .02 mm) and right (-.02, .02 mm) hemispheres, as well as bilateral adult cortical thickness (left = 0, .04 mm; right = -.01, .04 mm).

**Supplementary Figure 2.** Bland-Altman plots of pre- and post-edit global cortical thickness values in adolescent and adult groups. Results are presented by hemisphere. The x-axis represents the mean of pre- and post-edit cortical thickness values, and the y-axis illustrates the difference between these estimates.

**Results**

**Supplementary Table 1.** *Correlation between age and cortical thickness in adolescents*

| **#** | **FreeSurfer Region** | **Side** | **Cluster Size** | | **Peak MNI Coordinates** | | | **Peak Score** | **P*_corr_*** |
| --- | --- | --- | --- | --- | --- | --- | --- | --- | --- |
|  |  |  | **Vertices** | **Size (mm^2^)** | **X** | **Y** | **Z** | **T** |  |
| 1 | Supramarginal | L | 11221 | 5562.3 | -53.0 | -53.8 | 21.8 | -6.83 | .0002 |
| 2 | Superior frontal | R | 28635 | 15,143.21 | 10.4 | 50.3 | 36.1 | -6.11 | .0002 |
| 3 | Postcentral | L | 15717 | 7152.7 | -48.3 | -16.3 | 15.9 | -5.50 | .0002 |
| 4 | Pars triangularis | R | 4483 | 1857.5 | 35.3 | 28.6 | 6.5 | -5.45 | .0002 |
| 5 | Superior parietal | R | 1918 | 836.6 | 28.3 | -57.5 | 45.6 | -4.22 | .048 |
| 6 | Pericalcarine | L | 3135 | 1521.1 | -21.0 | -70.5 | 8.1 | -4.08 | .001 |
| 7 | Superior temporal | R | 2242 | 993.62 | 64.9 | -26.6 | 1.3 | -3.51 | .018 |
| 8 | Superior parietal | R | 2128 | 832.6 | 31.8 | -36.6 | 40.3 | -3.51 | .049 |
| 9 | Lingual | R | 1870 | 1035.8 | 24.6 | -47.2 | -6.7 | -3.02 | .014 |

Notes: p-values < .01 for Monte Carlo Null-Z simulations for multiple comparisons correction.

**Supplementary Table 2.** *Correlation between age and cortical thickness in adults*

| **#** | **FreeSurfer**  **Region** | **Side** | **Cluster Size** | | **Peak MNI Coordinates** | | | | **Peak Score** | **P*_corr_*** |
| --- | --- | --- | --- | --- | --- | --- | --- | --- | --- | --- |
|  |  |  | **Vertices** | **Size (mm^2^)** | **X** | **Y** | **Z** | **t** | |  |
| 1 | Pars triangularis | R | 3938 | 2128.0 | 54.3 | 26.2 | 8.0 | -5.07 | | .0002* |
| 2 | Precentral | R | 2283 | 1035.7 | 40.6 | 4.2 | 23.6 | -4.97 | | .009* |
| 3 | Caudal middle frontal | R | 2222 | 1190.7 | 34.4 | 6.9 | 56.0 | -4.57 | | .002* |
| 4 | Middle temporal | R | 3464 | 2063.2 | 45.6 | 9.9 | -35.9 | -4.36 | | .0002* |
| 5 | Middle temporal | L | 2485 | 1514.62 | -59.7 | -42.6 | -10.1 | -3.46 | | .001* |
| 6 | Insula | L | 16537 | 7645.6 | -32.9 | 1.6 | 13.5 | -3.13 | | .0002 |
| 7 | Paracentral | L | 11481 | 5627.8 | -13.3 | -21.4 | 46.6 | -2.61 | | .0002 |

Notes: p-values < .05, *p-values <.01 for Monte Carlo Null-Z simulations for multiple comparisons correction.

**Supplementary References**

Backhausen, L. L., Herting, M. M., Buse, J., Roessner, V., Smolka, M. N., & Vetter, N. C. (2016). Quality Control of Structural MRI Images Applied Using FreeSurfer-A Hands-On Workflow to Rate Motion Artifacts. *Frontiers in Neuroscience*, *10*, 558. https://doi.org/10.3389/fnins.2016.00558

Dale, A. M., & Sereno, M. I. (1993). Improved Localizadon of Cortical Activity by Combining EEG and MEG with MRI Cortical Surface Reconstruction: A Linear Approach. *Journal of Cognitive Neuroscience*, *5*(2), 162–76. https://doi.org/10.1162/jocn.1993.5.2.162

Desikan, R. S., Ségonne, F., Fischl, B., Quinn, B. T., Dickerson, B. C., Blacker, D., … Killiany, R. J. (2006). An automated labeling system for subdividing the human cerebral cortex on MRI scans into gyral based regions of interest. *NeuroImage*, *31*(3), 968–80. https://doi.org/10.1016/j.neuroimage.2006.01.021

Fischl, B., & Dale, A. M. (2000). Measuring the thickness of the human cerebral cortex from magnetic resonance images. *Proceedings of the National Academy of Sciences of the United States of America*, *97*(20), 11050–5. https://doi.org/10.1073/pnas.200033797

Fischl, B., Liu, A., & Dale, A. M. (2001). Automated manifold surgery: constructing geometrically accurate and topologically correct models of the human cerebral cortex. *IEEE Transactions on Medical Imaging*, *20*(1), 70–80. https://doi.org/10.1109/42.906426

Fischl, B., Sereno, M. I., & Dale, A. M. (1999). Cortical surface-based analysis. II: Inflation, flattening, and a surface-based coordinate system. *NeuroImage*, *9*(2), 195–207. https://doi.org/10.1006/nimg.1998.0396

Fischl, B., van der Kouwe, A., Desitrieux, C., Halgren, E., Segonne, F., Salat, D. H., … Dale, A. M. (2004). Automatically Parcellating the Human Cerebral Cortex. *Cerebral Cortex*, *14*(1), 11–22. https://doi.org/10.1093/cercor/bhg087

Ronan, L., Alexander-Bloch, A. F., Wagstyl, K., Farooqi, S., Brayne, C., Tyler, L. K., & Fletcher, P. C. (2016). Obesity associated with increased brain age from midlife. *Neurobiology of Aging*, *47*, 63–70. https://doi.org/10.1016/j.neurobiolaging.2016.07.010

Talairach, J., & Tournoux, P. (1988). Co-planar stereotaxic atlas of the human brain. 3-Dimensional proportional system: an approach to cerebral imaging.
